# Parallel comparison of Illumina RNA-Seq and Affymetrix microarray platforms on transcriptomic profiles generated from 5-aza-deoxy-cytidine treated HT-29 colon cancer cells and simulated datasets

**DOI:** 10.1186/1471-2105-14-S9-S1

**Published:** 2013-06-28

**Authors:** Xiao Xu, Yuanhao Zhang, Jennie Williams, Eric Antoniou, W Richard McCombie, Song Wu, Wei Zhu, Nicholas O Davidson, Paula Denoya, Ellen Li

**Affiliations:** 1 School of Medicine, Stony Brook University, Stony Brook, NY, 11794, USA; 2 Cold Spring Harbor Laboratory, Cold Spring Harbor, NY, 11724, USA; 3 Department of Applied Mathematics and Statistics, Stony Brook University, Stony Brook, NY, 11794, USA; 4 Department of Medicine, Washington University St. Louis, St. Louis, MO, 63110, USA

## Abstract

**Background:**

High throughput parallel sequencing, RNA-Seq, has recently emerged as an appealing alternative to microarray in identifying differentially expressed genes (DEG) between biological groups. However, there still exists considerable discrepancy on gene expression measurements and DEG results between the two platforms. The objective of this study was to compare parallel paired-end RNA-Seq and microarray data generated on 5-azadeoxy-cytidine (5-Aza) treated HT-29 colon cancer cells with an additional simulation study.

**Methods:**

We first performed general correlation analysis comparing gene expression profiles on both platforms. An Errors-In-Variables (EIV) regression model was subsequently applied to assess proportional and fixed biases between the two technologies. Then several existing algorithms, designed for DEG identification in RNA-Seq and microarray data, were applied to compare the cross-platform overlaps with respect to DEG lists, which were further validated using qRT-PCR assays on selected genes. Functional analyses were subsequently conducted using Ingenuity Pathway Analysis (IPA).

**Results:**

Pearson and Spearman correlation coefficients between the RNA-Seq and microarray data each exceeded 0.80, with 66%~68% overlap of genes on both platforms. The EIV regression model indicated the existence of both fixed and proportional biases between the two platforms. The DESeq and baySeq algorithms (RNA-Seq) and the SAM and eBayes algorithms (microarray) achieved the highest cross-platform overlap rate in DEG results from both experimental and simulated datasets. DESeq method exhibited a better control on the false discovery rate than baySeq on the simulated dataset although it performed slightly inferior to baySeq in the sensitivity test. RNA-Seq and qRT-PCR, but not microarray data, confirmed the expected reversal of *SPARC *gene suppression after treating HT-29 cells with 5-Aza. Thirty-three IPA canonical pathways were identified by both microarray and RNA-Seq data, 152 pathways by RNA-Seq data only, and none by microarray data only.

**Conclusions:**

These results suggest that RNA-Seq has advantages over microarray in identification of DEGs with the most consistent results generated from DESeq and SAM methods. The EIV regression model reveals both fixed and proportional biases between RNA-Seq and microarray. This may explain in part the lower cross-platform overlap in DEG lists compared to those in detectable genes.

## Background

In recent years, RNA-Seq emerged as an appealing alternative to classical microarrays in measuring global genomic expressions [1,2]. The RNA-Seq technology has been applied to many human pathological studies such as prostate cancer [3], neurodegenerative disease [4], retina defection [5], and colorectal cancer [6]. Gene detection in RNA-Seq, unlike microarray, is not dependent on probe design; rather it relies on short nucleotide reads mapping which can attain exceedingly high resolution. Furthermore, the RNA-Seq gene counts cover a larger dynamic range than microarray probe hybridization based design. On the other hand, microarray technology is still widely used because of lower costs and wider availability [7]. Previous studies comparing parallel RNA-Seq with microarray data have reported good correlation between the two platforms [1,8-13]. While classical correlation approaches can evaluate the strength of the association between the two platforms, they have been insufficient in gauging proportional and fixed biases between the two platforms. Given the uncertainties in measuring gene expressions for both platforms, we have therefore applied the Errors-In-Variables (EIV) regression model [14]. The EIV model is a more suitable regression method for this type of platform comparison because (1) it reflects measurement errors from both platforms, (2) its goodness-of-fit measure reflects the Pearson correlation, yet with the added advantages of (3) providing a measure for fixed bias and, a measure for proportional bias [15].

A major rationale for conducting global transcriptomic studies is to identify genes that are differentially expressed between two or more biological conditions. In previous comparisons of the differentially expressed gene (DEG) lists generated using parallel RNA-Seq and microarray data, the biological groups that were studied were often very different (e.g. liver vs. kidney, or malignant breast cell line vs. normal breast cell line) [1,8]. In the current study, parallel sets of RNA-Seq and Affymetrix microarray data were generated on a single HT-29 colon cancer cell line that was treated with and without 5-aza-deoxy-cytidine (5-Aza), a DNA methylation enzyme inhibitor. The concentrations of 5-Aza used in the present study (0 μM, 5 μM and 10 μM), approximated or exceeded the concentration previously reported to reverse hypermethylation of the *SPARC *(EMBL: ENSG00000113140) gene promoter and reverse suppression of *SPARC *mRNA expression in HT-29 cells [16]. In this study, paired ends 100bp RNA-Seq data was generated as opposed to single end RNA-Seq data described in similar reports [1,8,10,11,13]. Moreover, most of the previous studies comparing the two platforms were usually based on one or two DEG detection methods, which were relatively outdated or not inclusive [7,15,17]. Our study surveyed an array of currently used algorithms to identify DEGs in parallel for both microarray and RNA-Seq data. We sought to determine which pair of microarray and RNA-Seq algorithms would yield the largest overlap in the DEG lists under the same statistical significance level. A simulation study was further conducted using published parallel RNA-Seq and microarray datasets [1], to assess the consistency of different DEG methods across platforms and their ability in identifying true positives. Quantitative reverse transcriptase polymerase chain reaction assays (qRT-PCR) was used to assay expression of the *SPARC *gene and other DEGs selected by using 1) both datasets, 2) RNASeq data only and 3) microarray data only. Finally we determined which Ingenuity Pathways Analysis (IPA) canonical pathways were identified by 1) both datasets, 2) RNASeq data only and 3) microarray data only.

## Methods

### 5-Aza treatment of HT-29 cells

The HT-29 (ATCC) colon cancer cell line was maintained in DMEM supplemented with 10% fetal bovine serum, 1% kanamycin, streptomycin-penicillin, and incubated at 37° C and 5% CO2. Three replicative 150 mm cultures were treated with: 1) dimethyl sulfoxide (vehicle alone, 0 μM 5-Aza); 2) 5 μM 5-Aza and 3) 10 μM 5-Aza; for five days. These 5-Aza concentrations are similar and greater than the 5-Aza concentration previously reported to increase apoptosis, alter genome methylation as well as mRNA gene expression in HT-29 cells [16]. The HT-29 cells were washed with phosphate buffered saline on the plate prior to scraping and centrifuging the cells. Total RNA was extracted separately from each of these nine cultures using TRI Reagent according to the manufacturer's recommendations. The RNA quality was assessed using an Agilent 2100 Bioanalyzer (Agilent Tech., Palo Alto, CA) to have a RNA Integrity Number score *≥ *7. Each of the nine RNA samples was used to generate parallel RNA-Seq, microarray and qRT-PCR data.

### Illumina RNA-Seq data

Aliquots (1 μg) of nine RNA samples (triplicate samples for each of three experimental conditions), were subjected to paired-ends 100 bp Illumina sequencing. The RNA-Seq libraries were prepared and sequenced at Cold Spring Harbor Laboratories using the TruSeq RNA Sample Preparation Kit (Illumina Inc., San Diego, CA). In brief, mRNA was purified and fragmented, followed by cDNA synthesis with random hexamers. This product then underwent end repair, adapter ligation, and size selection using AMPure XP beads (Beckman Coulter Inc., Brea, CA) to isolate DNA templates of 320nt fragments and to remove excess adapters. The cDNA was PCR amplified. Each library was sequenced using Illumina 2000 sequencer (Illumina Inc., San Diego, CA) on 2 lanes of the flow cell. Between 41 and 88 million reads were generated for each of the RNA samples. The sequences were filtered using FASTX-Toolkit [18] to remove sequences with low Phred scores (~ first 3 nucleotides). The short reads fastq files were processed using Tophat (v2.0.1) [19] and mapped to the reference Ensembl human genome 19 using default settings for paired reads. Cufflink program (version 1.3.0) [20] or HTSeq-count (v 0.5.3p7) [21] were subsequently employed to convert aligned short reads (BAM format) into Fragments Per Kilobase of exon model per Million mapped fragments (FPKM) or raw gene counts. In the following step, a filter procedure was applied to remove gene entries with max alignment number of < 10 in all three replicates of the experimental groups (control or 0 μM, 5 μM and 10 μM 5-Aza). The RNA-Seq data were deposited in NCBI's Gene Expression Omnibus database with accession number GSE41588.

### Affymetrix microarray data

Aliquots (150 ng) of the same nine RNA samples were each labeled (single color), hybridized to Affymetrix hgu133plus2.0 (Affymetrix Inc., Santa Clara, CA) arrays, and the arrays were scanned in the Stony Brook University DNA Microarray Core Facility, according to the manufacturer's protocol. Note each RNA sample was hybridized to a separate microarray chip. Microarray data were preprocessed using Bioconductor's *affy *package followed by a custom filter procedure to retain the probe entries that were present in all three biological triplicates of one experimental group (control or 0 μM, 5 μM and 10 μM 5-Aza). RMA normalization was applied to scale the replicates to a comparable range. If multiple probes on the array corresponded to a single gene, the probe with the highest intensities was used to represent the gene intensity. The microarray data have been deposited in NCBI's Gene Expression Omnibus and are accessible through GEO Series accession number GSE41364.

### Platform comparison based on gene expression levels and correlations

General between-platform association analysis was applied to compare RNA-Seq with microarray data profiles. This includes a detectable gene determination for each group after the filter procedure, in which detectable genes were identified and compared respectively between the two platforms. In addition, the general gene expression profiles from RNA-Seq or microarray were examined in a scatter plot with Pearson and Spearman correlation coefficients calculated for all the genes (including those removed by the filtering procedure). Detectable genes which are RNA-Seq exclusive were compared to the overlapped ones using expression intensity histograms. This analysis was performed to verify the sensitivity of RNA-Seq technology in detecting genes expressed at low levels.

### Errors-In-Variables (EIV) regression model

Both normalized microarray data and RNA-Seq FPKM values were transformed into log2 scale and subsequently converted to unit-free ratios by dividing a pre-selected housekeeping gene, *ZNF311*(EMBL: ENSG00000197935). This gene was selected based on its moderate intensity and consistent expression levels (rank of expression intensity) across all samples on both platforms. We did not use *GAPDH *(EMBL: ENSG00000111640) as housekeeping gene because it is highly expressed. In our experiment a moderate expressed gene is more suitable as the reference for all measured genes.

We subsequently constructed a linear functional Errors-In-Variables (EIV) regression model based on the log2 scaled, normalized values between RNA-Seq (Y) and Microarray (X) as follows:

(1) $$\begin{array}{c} Y_{ij}=\alpha +\beta \xi_{i}+\in_{ij},\in_{ij}\sim N\left(0,\lambda \sigma_{\in }^{2}\right)\\ X_{ij}=\xi_{i}+\delta_{ij},\delta_{ij}\sim N\left(0,\sigma_{\delta }^{2}\right)\\ \lambda =\frac{\sigma_{\in }^{2}}{\sigma_{\delta }^{2}} \end{array}$$

Here Y_ij _denotes the normalized value of RNA-Seq expression for gene i and sample j and X_ij _represents the normalized microarray expression intensity. Moreover, $\xi_{i}$ is the expected value of Y; $\in_{ij}$ and $\delta_{ij}$ are independent platform measurement errors with mean zero and variances $\sigma_{\in }^{2}$ and $\sigma_{\delta }^{2}$. A prerequisite of this EIV model is the homoscedasticity assumption and in practice we removed the top 1% of genes with the largest variation and examined the remaining genes using Levene's test [22] to ensure equal error variances on both platforms. The ratio of error variances λ is estimable when we have multiple observations from the same sample, which we fortunately do in this study with 3 replicates per sample. When the errors are normally distributed we can obtain the point estimators of the model parameters via the maximum likelihood method [23]. The confidence intervals for the regression slope and intercept can be obtained via the bootstrap resampling method.

In our study, an EIV regression model was constructed for each of the three experimental HT-29 cell groups (control or 0 μM, 5 μM, and 10 μM 5-Aza) and the R rootSolve package (v. 1.6.3) was used to compute the point estimators for each regression model. The bootstrap resampling method with 1000 times resampling were performed to derive the corresponding 95% confidence interval for the regression intercept α and the regression slope ß as an estimate of the fixed and the proportional bias respectively. Statistically, the confidence interval of α covering 0 indicates an absence of fixed bias; whereas the confidence interval of ß encompassing 1 implies the absence of proportional bias.

### DEG algorithms for microarray and RNA-Seq data

The T-test with Benjamini-Hochberg correction [24], SAM [25] and eBayes [26] algorithms were applied to the filtered Affymetrix microarray data to generate DEG lists ( > 2-fold, FDR ≤ 0.05) for the following two pair-wise comparisons: 1) 5µM vs. 0µM 5-Aza groups and 2) 10µM vs. 5µM 5-Aza groups, respectively. The Cuffdiff [20], SAMSeq [27], DESeq [28], baySeq [29] algorithms were applied to the filtered RNA-Seq data to generate DEG lists based on the same cutoff (> 2-fold or <0.5, FDR ≤ 0.05). NOISeq [30] was applied to the RNA-Seq data and the DEG list was subsequently filtered for a threshold of (> 2-fold or <0.5). The popular edgeR algorithm [31] was not included since it closely resembled the DESeq algorithm [28].

### Comparing DEG algorithms using simulated data

In our simulation study, we designed a simulation method which generated consistent RNA-seq and microarray data in comparing DEG algorithms of the two platforms. The RNA-Seq and microarray simulations were built upon parallel RNA-Seq and microarray datasets reported previously by Marioni et.al. (GSE11045) [1]. For this simulated analysis we could not apply the Cuffdiff algorithm because the previously published RNA-Seq data was reported only as raw gene counts without exon level information.

Assessment of error in the microarray data was conducted using the model described previously by Rocke and Durbin [32] in which observed gene expression is modeled as $\text{y}=\alpha +\text{u}{\text{e}}^{\eta }+\varepsilon$, where η, ε are normal error term and u is true intensity. A variance stabilizing transformation was applied as previously described [33] as shown in equation (2):

(2) $$\text{g}\left(y\right)=\text{ln}\left(\text{y}-\alpha +\sqrt{{\left(\text{y}-\alpha \right)}^{2}+\text{c}}\right)$$

Here y is the probe expression intensity, α denotes the mean of background noise, c stands for $\text{sd}\left(\varepsilon \right)/\text{sd}\left({\text{e}}^{\eta }\right)$; η and ε are normally distributed error terms. In our simulation, RMAexpress [34] was used in noise correction, the genes expressing at the bottom 1% level were used to estimate sd(ε), and the log transformation of genes expressing at the top 1% level (after correction for noise) were used to estimate $\text{sd}\left(\eta \right)$. The mean of background noise --- α, was estimated by subtracting from the uncorrected mean intensity of the genes expressing at the bottom 1% level, the noise corrected mean intensity of the genes expressing at the bottom 1% level. It is easy to solve that:

$$\text{y}-\alpha =\text{u}{\text{e}}^{\eta }+\varepsilon =\frac{e^{2g\left(y\right)}-c}{2e^{g\left(y\right)}}$$

By averaging across the $ln\left[\left(e^{2g\left(y\right)}-c\right)/2e^{g\left(y\right)}\right]$, we can approximately eliminate the effect of η and *ε*; and the transformed data could then be used to build an empirical distribution of u. The true expression levels of simulated genes were sampled from this empirical distribution in such a way that: a histogram of true data was generated using 500 bins at first step; a simulated gene was then assinged to a bin based on the frequency with a small turbulance added to its value. Uniform distribution (ranging the length of the bin) was assumed to the turbulance term to differentiate genes in the same group. As a result, the transformed expression level of a gene at a certain x% quantile of a given sample is equal to the same x% quantile of g(y) in terms of distribution + a small turbulance. The turbulance added same effect of variation to every gene because of the variance stablizing transformation.

The RNA-Seq data were fitted in a negative binomial model as described by Kvam [35]. The mean expression level λ was sampled from a gamma distribution whose parameters were determined by fitting the true data with maximum likelihood method; similarly, the over-dispersion parameter *φ *was also generated from a gamma distribution described before [29]. In practice, the distribution of λ was slightly rescaled to the range of the real data. Subsequently, we sampled both microarray g(y) and RNA-Seq λ of 10,000 genes from each corresponding distribution with a strict rule on quantile consistency (any gene of the α percentile of one distribution shall have the same quantile in the other dataset). In reality, sample percentile converge to distribution percentile when sample size is large, therefore a Spearman correlation of 1 was approximated in our simulated data sets across platforms.

Pre-defined significant DEGs were randomly sampled so that the log fold changes of these preset DEGs were generated from a mixed normal distribution where the probabilities of being up and down regulated were both equal. Moreover, in our analysis, the absolute expectation of log fold changes and standard errors for up and down regulated genes were set to be the same.

In practice, we generated 10 sets of simulated data in practice with 5 replicates included in both treatment and control group and 1000 random selected genes were preset to be differently expressed in different levels using a method of 95% minimum fold change such that the 1000 preset *bona fida *DEGs were generated with their fold changes following a log-normal distribution with 95% of the 1000 genes having their fold changes above the given level. We implemented a total of seven algorithms in our study, namely T test, SAM and eBayes on microarray data and baySeq, DESeq, SAMseq, NOISeq for RNA-Seq data.

In this work, the sensitivity and false discovery rates (FDR) were firstly evaluated for each DEG method under the 95% minimum fold change of 2 for preset DEGs and FDR cutoff of 0.05. For NOISeq method, a q = 0.8 (recommended by NOISeq author) criterion was used due to the absence of FDR control in this method. We further evaluated the sensitivity and false positive rate of each DEG algorithm by varying the differential significance levels of the preset 1000 genes using 95% minimum fold change method. Specifically, a range of values from 0.5 to 4 by an increment of 0.5 were used to generate the simulated DEGs.

### qRT-PCR analysis of control HT-29 RNA samples vs. 5 μM 5-Aza treated HT-29 RNA samples

Reverse transcription was performed on 5 μg aliquots of each of the six RNA samples using the SuperScript III First-Strand Synthesis System for RT-PCR (Invitrogen Life Technologies, Grand Island, NY) according to the manufacturer's protocol. QRT-PCR was initially performed on serial dilutions of the cDNA for each Taqman assay kit using Taqman assay kits (Invitrogen Inc., Carlsbad, CA) in order to confirm that each of the assays were conducted in the linear range and the slopes of the threshold cycle Ct when plotted against the dilution were the same for all of the assays. Thirteen genes were selected by the majority vote of platform specific DEG detection methods and are categorized into 3 groups, which are: 1) commonly identified on RNA-Seq and microarray datasets; 2) RNA-Seq data only and 3) microarray data only. This list of genes, with the additional *SPARC *and *GAPDH *(EMBL: ENSG00000111640), and the corresponding commercial Taqman assays are listed in Additional file 1. The qRT-PCR assays were conducted in triplicate for each RNA sample. The ΔCt values (Ct for *GAPDH *- Ct for the test gene) were calculated for each RNA sample. The Student t-test was used to analyze whether there was a significant difference between the mean ΔCt for the control vs. the 5 μM 5-Aza treated HT-29 groups, with a threshold significance level of 0.05. The fold change in gene expression was calculated as 2^-ΔΔCt ^(ΔΔCt = ΔCt of 5-Aza group - ΔCt of control group).

### Ingenuity Pathway Analysis of microarray and RNA-Seq data

Based on the results of the simulation, we performed IPA analysis (Ingenuity^® ^Inc, Redwood city, CA) on up-regulated DEGs (5 µM vs. 0 µM 5-Aza) and down-regulated DEGs respectively. 5 DEG lists were generated by the SAM, eBayes, Cuffdiff, DESeq and baySeq algorithms. Significantly enriched canonical pathways were selected based on the p value cutoff of 0.05 and included gene number > 3 [36].

## Results

### General association between the two platforms

**Figure 1:**
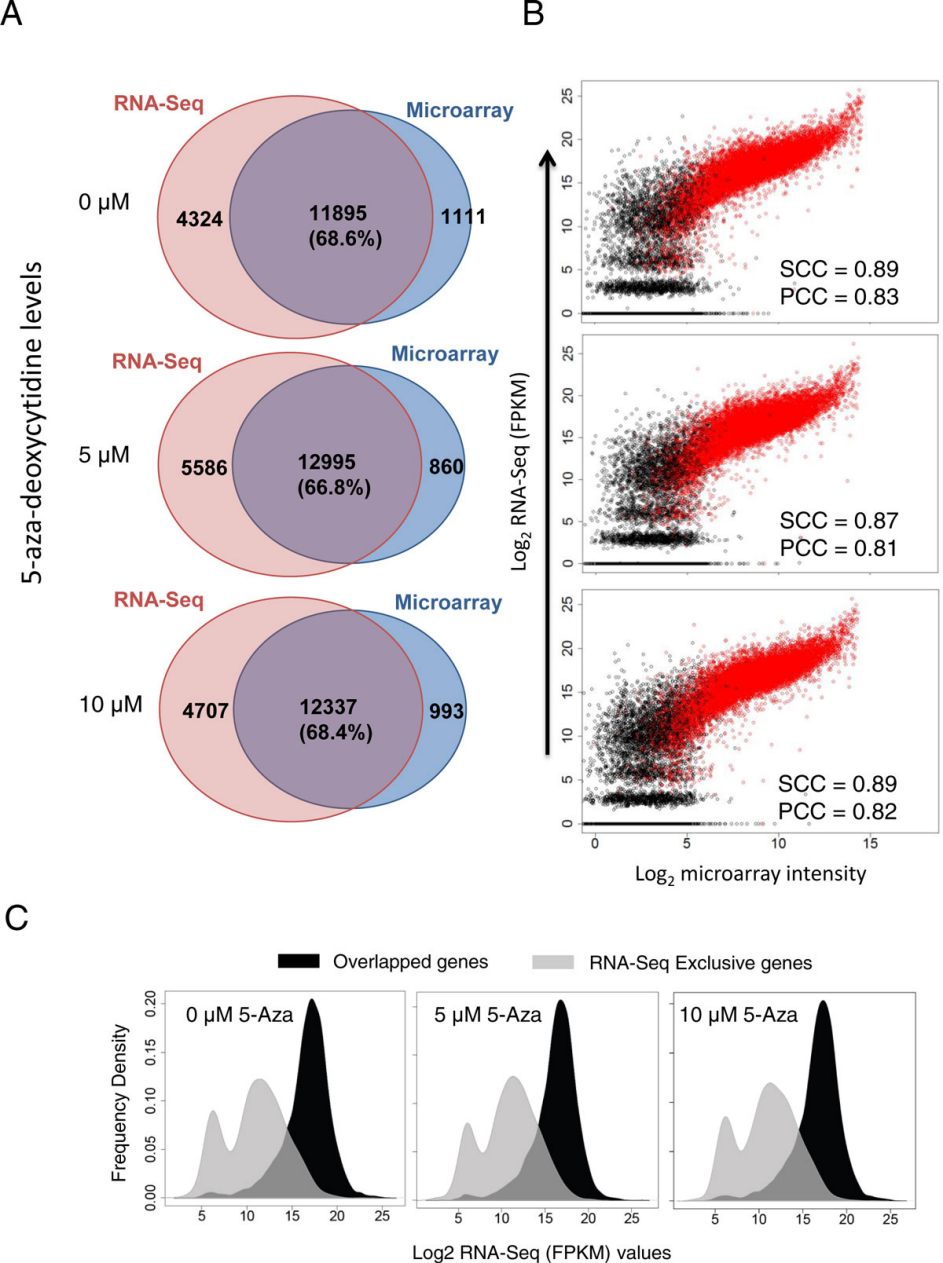
**Expressional consistency between RNA-Seq and microarray data**. **A**. Detectable genes reported by each technology based on a common filter procedure (**See Methods**). Venn diagrams of detectable genes are shown 3 experimental conditions (0 µM, 5 µM and 10 µM) respectively, and overlap rates are calculated by dividing number of commonly detectable genes by the union. **B**. By-group scatter plot depicting the expression profiles of all genes. Log2 transformed FPKM values from RNA-Seq and log2 scaled microarray gene intensities (normalized) are used in the scatterplot. We added 1 to FPKM value before log2 transformation to facilitate calculation. Commonly detected genes are shown in red color while platform exclusive genes are denoted in black. Both Pearson correlation coefficients (PCC) and Spearman correlation coefficients (SCC) were calculated based on all gene entries (except for those not having probe names on the array or RNA-Seq reference genome). **C**. By-group expressional density histogram for both commonly detectable genes and RNA-Seq specific ones. The *x*-axis denotes the RNA-Seq FPKM value (log2 scale) distribution and *y*-axis shows the frequency of genes within each category. Commonly detectable genes are depicted in black while RNA-Seq exclusive genes are shown in grey color.

A total of 13006, 13855 and 13330 genes were detected respectively for the 0µM, 5 µM and 10 µM 5-Aza HT-29 microarray datasets, whereas 16219, 18581 and 17044 genes were identified on RNA-Seq for the 3 groups. On average, the Illumina RNA-Seq detected ~29.0% more genes than its microarray counterpart and a significant portion (~22.1%) of the RNA-Seq specific genes did not have corresponding probe sets on the array. The overlap rates of the genes detected by both RNA-Seq and microarray datasets for the 0 μM, 5 μM and 10 μM 5-Aza HT-29 cultures, respectively, ranged between 66.8-68.6% (Figure 1A). We further profiled the expression pattern of all genes from both platforms and observed a general linear relationship between the two data sources. Both Pearson and the Spearman correlation coefficients were evaluated for each group and the results (Pearson correlation r = 0.81 ~ 0.83, Spearman correlation r_s _= 0.87 ~ 0.89, P value << 1×10^-10^) indicated a strong correlation between the two platforms (Figure 1B). This result is by and large consistent with previous reports in similar comparative settings [1,9-11,13]. We further examined the widely-reported sensitivity advantage of RNA-Seq over microarray platform. Group-wise density histograms were generated to examine the distribution of the commonly detectable genes and those having corresponding probes on the array yet are exclusively identified by RNA-Seq (Figure 1C). The histogram clearly showed disparate peaks between the two categories of genes with the overlapped ones forming a higher peak at the upper level of the expression scale and the microarray bereft genes mainly distributed at the lower end of the axis. This observation indicates that RNA-Seq may be superior to the microarray in detecting genes expressed at low levels.

### Applying EIV model for platform comparison

**Figure 2:**
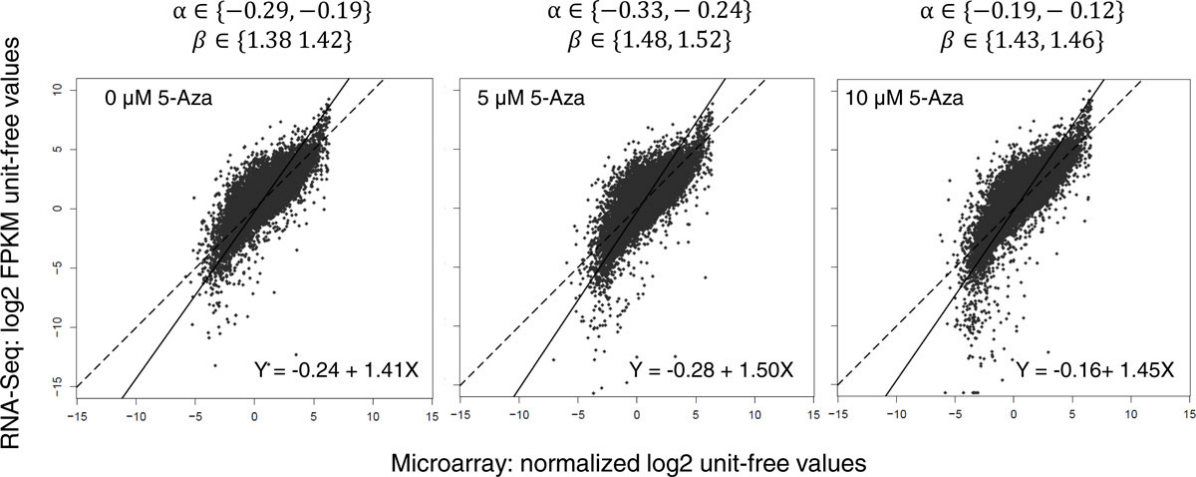
**EIV Regression Model Comparing Microarray and RNA-Seq Gene profiles**. EIV regression model is constructed for independent variable (microarray normalized gene intensities in log2 unit-free scale) and dependent variable (RNA-Seq FPKM values in log2 unit-free scale) for each of the experimental groups (5 µM, 10 µM and 0 µM) of HT29 samples. Log2 scaled unit-free normalized gene intensities are shown as grey circles in the scatter plot and EIV regression line is drawn in bold black. For each of the plots, a dashed reference line of Y = X (corresponding to perfect platform agreement) is also included to indicate the deviation of the real regression line from the reference. The estimated regression equation is shown in the lower-right section of each plot. The 95% bootstrap confidence interval for the regression intercept and slope (α and ß) are shown on the top of each plot.

An Errors-In-Variables regression model was built to investigate the consistency between normalized microarray gene abundances and the normalized FPKM genomic intensities from RNA-Seq platform with both measurements in log2 scale. Using the maximum likelihood estimation of the EIV model, we obtained a linear relationship of the gene expression profiles between RNA-Seq and microarray for each experimental group (Figure 2). In each regression model, the variance ratio λ was calculated numerically and the optimal value was used to determine the slope and intercept of the corresponding regression line. Based on the observation across all 3 groups, we found that the estimated fixed bias $\hat{\alpha }$ ranging from -0.12 to -0.33 with the corresponding 95% bootstrap confidence intervals for α not covering 0, indicating the existence of the fixed bias of measurements between the two platforms. Moreover, a clear deviation from the regression model and the reference Y = X line was observed (Figure 2). The estimated regression slope $\hat{\beta }$, representing the proportional bias, ranged from around 1.38~1.52, with the corresponding 95% bootstrap confidence intervals for β excluding 1 indicating the presence of proportional bias between the two platforms as well. This infers that the changes of microarray measured gene expression at per unit level do not equate to the same level of unit change on the RNA-Seq platform, a result possibly arising from the different signal quantification mechanisms between the two technologies (short reads counts versus fluorescence intensity).

### Comparison of DEG algorithms applied to experimental microarray and RNA-Seq HT-29 data

Three microarray DEG algorithms (T-test, SAM, eBayes) and five RNA-Seq algorithms (Cuffdiff, SAMSeq, DESeq, baySeq and NOISeq) were applied to the experimental HT-29 microarray and RNA data, respectively (See Additional file 2). The threshold was set at fold-change > 2 or less than 0.5 and a false discovery rate ≤ 0.05 for all of the eight algorithms except NOISeq. Since setting a fold change was not an option for NOISeq, we set a threshold of q = 0.8 and then subsequently filtered the selected genes with a threshold of fold-change > 2 or less than 0.5. Treatment of HT-29 cells with 5 μM 5-Aza (compared to the control HT-29 cells) resulted in up-regulation (↑) and down-regulation (↓) of genes. The T-test identified 392↑ 148↓, SAM identified 794↑ 256↓ and eBayes identified 782↑ 259↓ using the same microarray data (~13,000 detectable genes). Cuffdiff found 1149↑ 558↓, SAMSeq found 2262 ↑ 282↓, DESeq found 1840↑ 300↓, baySeq found 2013↑ 293↓, and NOISeq identified 673↑ 151↓ using the same RNA-Seq data (~17,000 detectable genes). All of the algorithms demonstrated an overall upregulation of gene expression after treatment of 5 μM 5-Aza. This is consistent with the concept that 5-Aza treatment reverses hypermethylation of gene promoters in HT-29 colon cancer cells and thus activates corresponding genes. However, activation of *SPARC *gene expression, which was previously reported after treatment of HT-29 cells with 4 μM 5-Aza [16], was observed in the RNA-Seq data only, and not in the microarray data.

The effect of increasing the concentration of 5-Aza from 5 μM to 10 μM 5-Aza was also analyzed using the eight algorithms and the same threshold parameters. The T-test identified 0↑ 2↓, SAM identified 13↑ 285↓ and eBayes identified 41↑ 278↓ using the same microarray data (~13,000 detectable genes). Cuffdiff detected 15↑ 485↓, SAMSeq detected 0↑ 626↓, DESeq detected 43↑ 389↓, baySeq detected 58↑ 424↓, and NOISeq detected 95↑ 123↓ using the same RNA-Seq data (~17,000 detectable genes). With the exception of the T-test and NOISeq, the remaining six algorithms detected an overall down-regulation in gene expression when the concentration of 5-Aza was increased from 5 μM to 10 μM. This could reflect toxic effects of 5-Aza at the higher 10 μM concentration.

The cross-platform overlap rates between the DEG lists generated by each of the three microarray algorithms with DEG lists generated by each of the five RNA-Seq algorithms are summarized in Table 1. The highest cross-platform overlap rates were achieved by comparing the baySeq and DESeq generated DEG lists using the RNA-Seq data, with the SAM and eBayes generated DEG results using the microarray data.

**Table 1:** Cross-platform overlap in DEG lists using RNA-Seq and microarray HT-29 data.

Comparison (5-Aza)	Methods	Cuffdiff	SAMSeq	baySeq	DESeq	Noiseq
5 µM vs 0 µM	T-test	25.8%	22.5%	24.5%	25.8%	29.7%
	SAM	39.9%	39.9%	**42.7%**	**44.5%**	33.5%
	Ebayes	39.5%	39.5%	**42.2%**	**44.0%**	33.3%

10 µM vs 5 µM	T-test	0.5%	0.5%	0.6%	0.6%	0.0%
	SAM	31.1%	30.2%	32.6%	34.2%	19.4%
	Ebayes	30.3%	28.3%	31.9%	33.5%	19.1%

The cross-platform overlap rates (%)^1 ^were calculated between the three microarray DEG algorithms (T-test, SAM, eBayes) and the five RNA-Seq algorithms (Cuffdiff, SAMSeq, baySeq, DESeq, and NOISeq) as described in **Methods**. Comparisons were made to 1) measure the effect of treating HT-29 cells with 5 μM 5-Aza compared to control cells and 2) the effect of increasing the 5-Aza concentration from 5 μM to 10 μM.

¹The DEG overlap rates were calculated by dividing the number of overlapped DEGs over the union number of DEGs identified from both methods. Overlap rates higher than 40% are denoted in bold style.

### Comparison of DEG algorithms applied to simulated microarray and RNA-Seq data

Simulated datasets were generated from independent parallel RNA-Seq and microarray datasets generated from kidney tissue [1]. In this experiment, technical rather than biological replicates were used to generate the data set. It was not feasible to evaluate Cuffdiff using this method since the data set only provided gene counts without exon level information. The overlaps in the DEG lists are summarized in Table 2. To be consistent with the thresholds applied when these algorithms were applied to the experimental HT-29 data, we used the 95% minimum fold change method with FC level = 2 on preset positives and FDR ≤ 0.05 for each algorithm (**See Methods**). Intra-microarray platform comparisons revealed that the T-test generated DEG list overlapped poorly with both the SAM and the eBayes generated DEG lists. However, SAM and eBayes DEG lists achieved 95% overlap with each other. Intra-RNA-Seq platform comparisons revealed that baySeq and DESeq DEG lists achieved 75.7% overlap with each other, while the overlap percentages ranged between 46% and 54% for the remaining RNA-Seq algorithms. The highest cross-platform overlap percentages (48-50%) were observed between the SAM and eBayes microarray DEG lists and the baySeq and DESeq RNA-Seq DEG lists. Not surprisingly, the T-test DEG list overlapped poorly with the results of all the RNA-Seq algorithms.

**Table 2:** Intra- and cross-platform comparison of DEG lists generated from simulated microarray and RNA-Seq data.

	T-test	Ebayes	SAM	baySeq	DESeq	SAMseq	NOISeq
T-test	100.0%						
Ebayes	5.1%	100.0%					
SAM	4.8%	95.1%	100.0%				
baySeq	**3.0%**	**48.1%**	**49.6%**	100.0%			
DESeq	**3.3%**	**48.7%**	**50.2%**	75.7%	100.0%		
SAMseq	**3.4%**	**36.5%**	**37.0%**	54.1%	51.9%	100.0%	
NOISeq	**3.3%**	**39.7%**	**39.7%**	46.5%	50.4%	46.9%	100%

The simulations were carried out as described in **Methods**. The overlap percentages was calculated based on the percent of true positive DEGs (95% minimum fold change method: FC ≥ 2 & FDR < = 0.05) identified by each of the three microarray algorithms (T-test, SAM, eBayes) and four RNA-Seq algorithms (SAMseq, baySeq, DESeq, NOISeq).

¹The overlap rate was calculated based on true positive DEGs called by each method. The microarray and RNA-Seq cross-platform DEG overlap rates are shown in **bold **style.

**Figure 3:**
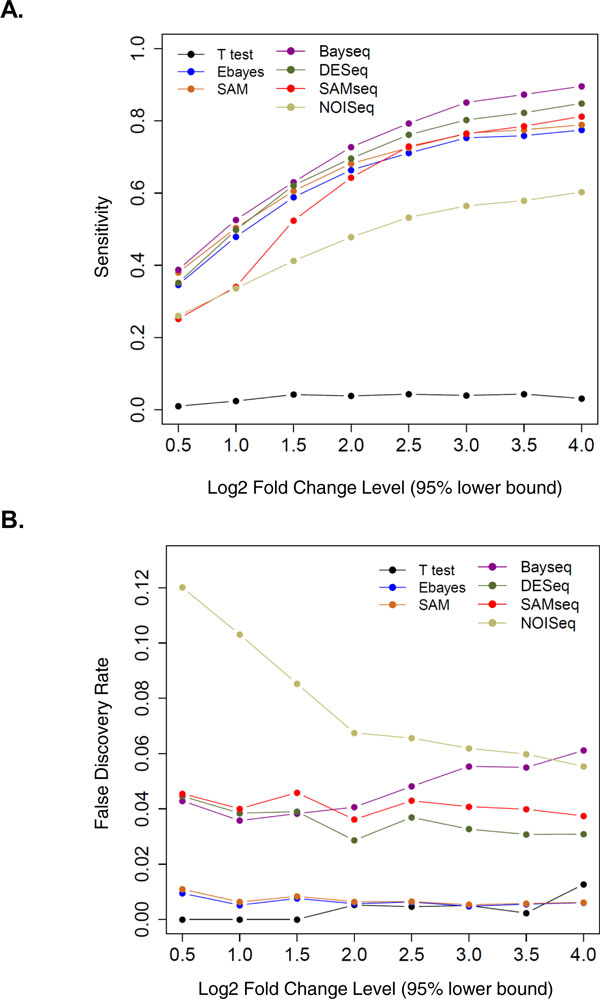
**Sensitivity and False Discovery Rate (FDR) curve plots for simulated data using each DEG method**. Sensitivity (**A.) **or FDR (**B**.) are calculated for 4 RNA-Seq DEG methods (SAMSeq, baySeq, DESeq, and NOISeq) and 3 microarray DEG algorithms (T-test, SAM, eBayes). Method curves are shown in different colors (see figure legends) at each 95% minimum fold change level for pre-determined DEGs. Each fold change on x-axis (in log2 scale) corresponds to the lower 5% fold change of normally distributed DEGs predefined in the simulation process (**See Methods**).

The sensitivity and the false discovery rate of each method were also calculated in ten simulated runs for the sake of accuracy evaluation. Based on the same significance level (95% minimum fold change method: FC cutoff ≤ 2 and FDR ≤ 0.05), we found that baySeq produced the highest sensitivity (52.6%) from RNA-Seq while SAM achieves the best sensitivity (50.4%) among microarray methods (Figure 3A). On the other hand, the RNA-Seq DEG algorithms generally result in higher FDRs (0.03~0.12) than their microarray counterparts (< 0.01). A further simulation test was conducted by changing the significance level of preset true positives. We observed that with the increase of true positive fold change (**See Methods**), the baySeq method continues to outperform other algorithms while DESeq, slightly inferior to baySeq, has been generally yielding good results, too (Figure 3A). On the microarray side, the SAM constantly achieves higher sensitivity than Ebayes and t-test. As per FDR evaluation, NOISeq method performed the worst among the four on FDR evaluation curve, particularly at the lower fold change end (Figure 3B, **Right**); The baySeq method, albeit more sensitive in calling true positives, has relatively poorer performance in controlling FDR and this drawback becomes more remarkable at higher fold change end (Figure 3B, **Left**). The specificity of each method was also evaluated and all of them were well above 99.9%.

### qRT-PCR Results

We applied yet a third technology, qRT-PCR, to confirm DEGs identified by the various microarray and RNA-Seq algorithms (Table 3). The *SPARC *gene expression was previously reported to be undetectable in control HT-29 cells but detectable in 4 μM 5-Aza treated HT-29 cells using a qualitative gel-based RT-PCR method [16]. We therefore conducted qRT-PCR assays on the control and 5 μM 5-Aza treated groups in this study on a selected subset of DEGs, including the *SPARC *gene (Table 3). Reversal of suppression of the *SPARC *gene was confirmed by qRT-PCR results since no *SPARC *gene expression was detected in any of the three control HT-29 RNA samples, but was detected in all three of the 5 μM 5-Aza treated HT-29 samples on RNA-Seq platform (Table 3). Overall qRT-PCR confirmed 75% of the DEGs identified by both RNA-Seq and microarray data, 66% of the DEGs identified by only by RNA-Seq data and 25% of the DEGs identified only by microarray data.

**Table 3:** Confirmation of DEGs selected using both RNA-Seq and microarray data, RNA-Seq only and microarray only by qRT-PCR.

Selected by^1^	Gene symbol	FC^2 ^qPCR	P value^3^	FC^2 ^microarray	FC^2 ^RNA-Seq
**BOTH**	**ALDH3A1**	**0.5**	**0.046**	**0.4**	**0.3**
BOTH	TGM2	1.6	0.762	0.4	0.3
**BOTH**	**IL8**	**3.2**	**0.002**	**3.7**	**3.4**
**BOTH**	**IL1R1**	**2.6**	**0.033**	**4.5**	**3.9**
Array	IRF7	0.5	0.068	2.0	1.4
**Array**	**TAF11**	**1.8**	**0.045**	**1.7**	**1.5**
Array	PLCL1	1.2	0.469	0.7	0.8
Array	LIPE	1.5	0.565	0.6	0.5
**SEQ**	**GGT1**	**6.1**	**0.043**	**1.0**	**2.6**
SEQ	GGT7	4.8	0.077	1.7	2.9
**SEQ**	**MAPK10**	**7.1**	**0.013**	**2.1**	**3.3**
**SEQ**	**RPSA**	**0.5**	**0.009**	**1.7**	**0.4**
SEQ	EFNB1	2.0	0.605	0.8	0.4
SEQ	SPARC^4^	INF	<<0.001	0.8	INF

The predicted polarity of the fold change for each DEG is listed along with the qRT-PCR calculated fold change (2^-ΔΔCt^). The P-values were calculated based on applying the T-test to ΔCt values of the control cells in comparison with the ΔCt values of the 5 μM 5-Aza treated cells (**see Methods**).

^1^Genes confirmed by RT-PCR as DEGs are marked in bold style (SEQ: Illumina RNA-Seq platform; Array: Affymetrix microarray platform)

^2^FC (fold change) is calculated as the group average of 5µM (5-aza-deoxycytidine)/Control, the normalized expression intensity value is used for microarray data while FPKM values from Cufflinks program are used for RNA-Seq data.

^3^P value is calculated using unpaired t-test on qPCR data; Genes that are significantly different between two groups are highlighted in bold style.

^4^The *SPARC *gene (not part of the 13 genes list) was specially added into the qPCR experiment for validation. On both qPCR and RNA-Seq platforms, its expression values in control group are all found to be zeros (below detection cutoff) whereas there are moderate expressions in 5µM group, therefore we consider the fold change as infinite positive denoted by INF with infinite minimal p values.

### Biological function analysis of DEG lists generated by microarray and RNA-Seq data

As shown in the outcome of the IPA analysis we performed (see Additional file 3), the overlap rate for the IPA canonical pathways selected by SAM and eBayes (microarray algorithms) was 81.4% (35 of 43 pathways); the overlap rate between the IPA canonical pathways was 52.1% (150 of 288 pathways) for DESeq and Cuffdiff, 91.4% (202 of 221 pathways) for DESeq and baySeq, and 48.0% (143 of 298 pathways) for baySeq and Cuffdiff. This is consistent with the observation that Cuffdiff DEGs had a lower overlap rate with either DESeq (56.5%) or baySeq (52.9%), while DESeq and baySeq has an overlap rate at 91.8%. Based on this observation; we compared cross-platform canonical pathways using the two microarray algorithms, SAM and eBayes, and the two RNASeq algorithms, DESeq and baySeq. All four of these algorithms identified 33 canonical pathways (See Table 4). 152 canonical pathways were identified only by the two RNASeq algorithms, DESeq and baySeq. No canonical pathways were identified only by the two microarray algorithms.

**Table 4:** Pathways commonly detected by SAM, eBayes, DESeq and baySeq

Up-regulated	
Atherosclerosis Signaling	Human Embryonic Stem Cell Pluripotency
Interferon Signaling	Bladder Cancer Signaling
LPS/IL-1 Mediated Inhibition of RXR Function	Activation of IRF by Cytosolic Pattern Recognition Receptors
Antigen Presentation Pathway	Factors Promoting Cardiogenesis in Vertebrates
Role of BRCA1 in DNA Damage Response	Role of CHK Proteins in Cell Cycle Checkpoint Control
Hepatic Fibrosis/Hepatic Stellate Cell Activation	FXR/RXR Activation
Type I Diabetes Mellitus Signaling	Glutathione-mediated Detoxification
Estrogen-mediated S-phase Entry	Hereditary Breast Cancer Signaling
GADD45 Signaling	Neuroprotective Role of THOP1 in Alzheimer's Disease
Caveolar-mediated Endocytosis Signaling	JAK/Stat Signaling
Graft-versus-Host Disease Signaling	Protein Ubiquitination Pathway
LXR/RXR Activation	PI3K/AKT Signaling
Oncostatin M Signaling	CDK5 Signaling
Autoimmune Thyroid Disease Signaling	Role of IL-17A in Arthritis
Cell Cycle Regulation by BTG Family Proteins	Aryl Hydrocarbon Receptor Signaling
ATM Signaling	Role of Osteoblasts, Osteoclasts and Chondrocytes in Rheumatoid Arthritis

**Down-regulated**	

FXR/RXR Activation	

## Discussion

In order to evaluate the performance of paired-end RNA-Seq data with a widely used commercial microarray platform, we chose to generate parallel datasets in a well-characterized experimental system, treatment of HT-29 colon cancer cells with 5-Aza, a DNA methyltransferase inhibitor [16,37]. The 5-Aza concentrations were chosen to approximate and exceed the concentration previously reported to increase apoptosis and alter genome methylation as well as mRNA gene expression in HT-29 cells [16]. Specifically reversal of hypermethylation of the *SPARC *promoter and reversal of suppression of *SPARC *gene expression were reported [16]. The RNA-Seq technology is rapidly advancing, hence paired-end rather than single end RNA-Seq data were generated for this study.

We first examined the detection sensitivity for both platforms. RNA-Seq detected more genes than microarray, particularly among genes expressed at low levels. This observation is consistent with previous studies [11,38]. The higher sensitivity of RNA-Seq can be attributed to its detection mechanism based on single-read/nucleotide resolution [39]. The microarray gene quantification results largely depend on the accuracy of probe fluorescence scanning; background signal and other confounding factors (e.g. stains on array surface) may conceal the real genetic signal for a probe having a low abundance. In this perspective, the difference in detection mechanism confers a natural advantage to RNA-Seq comparing to microarray. The genomic ranges covered by both platforms also differ significantly. In addition, RNA-Seq detects all sequences that are expressed and basically surveys all the known genes provided by hg19 reference genome (N = 23,368), whereas microarray only examines genes based on the predesigned probe sets included on the array (N = 18,209). The correlation analysis confirmed strong general concordance on the gene expression measurements across platforms. Both Pearson and the Spearman correlation coefficients between the two technologies were found well above 0.8 with P values << 0.001 indicating the data were in comparable quality to previously reported parallel microarray and RNA-Seq datasets [1,11,40]. Furthermore, the EIV regression model was applied since the classical correlation based analysis is insufficient in gauging the quantitative concordance of the two platforms and the existence of random errors in both measurements rendered the traditional ordinary least regression method unsuitable in the current case. As per our study, the EIV regression revealed the existence of both fixed and proportional biases between the microarray and RNA-Seq platforms. We found that the fixed bias plays a minor part while the proportional bias is the major source of discrepancy between the two platforms (Figure 2). Basically, an estimated fixed bias at -0.24 on the log2 scale reflected a trivial baseline difference, whereas an estimated ~1.45 proportional bias meant that a unit change on microarray gene intensity on the log2 scale corresponded to about 1.45 units change for RNA-Seq on the log2 scale. This regression model is consistent with the observation that RNA-Seq was more sensitive and exhibited a larger dynamic range than its microarray counterparts in measuring the expression level of the same transcript.

Since the major goal of conducting global transcriptomic studies is to identify genes that are differentially expressed between two or more biological groups, this study applied several DEG algorithms designed for either microarray or RNA-Seq data. Two of the most widely used microarray DEG algorithms in recent years, SAM and eBayes, are included in this study. The classical T-test, which is known to perform relatively poorly in microarray analysis was also evaluated as a "control" method [41]. While microarray data produces a continuous intensity, which commonly follows a log-normal distribution [42], the RNA-Seq gene expression level is discrete or digital in nature. The microarray DEG algorithms are based on continuous distribution of random variables (after log transformation of the probe hybridization intensities). On the other hand, RNA-Seq DEG algorithms are rapidly evolving. The earlier studies mostly relied on algorithms assuming a Poisson distribution on the gene counts [1,13,39] while the more recent methods utilized a negative binomial model which was considered better than Poisson assumption in explaining biological variability of the RNA-Seq data [28,43]. This study considers several of the currently used, popular RNA-Seq DEG algorithms: Cuffdiff, baySeq and DESeq which are roughly based on the negative binomial modeling of RNA-Seq data and the nonparametric SAMSeq and NOISeq methods, which are relatively model-free. Each of the methods has its own virtue and relevance: the Cuffdiff method is built to incorporate biological variability information (e.g. isoforms and fragment assignment uncertainty) from the initial short reads input. In baySeq algorithm, the estimate of significance is based on an empirical Bayes approach, which ranks the DEGs by posterior probabilities of the treatment group. DESeq assumes a locally linear relationship between variance and mean expression level. The SAMSeq algorithm, on the other hand, differs from the aforementioned algorithms by identifying DEGs using a Wilcoxon rank based nonparametric approach, which is relatively free from model biases. Lastly, the NOISeq algorithm evaluates the log-ratio of normalized counts (M value) versus their absolute difference (D value) and determined their differential significance by comparing to the noise distribution, and is designed to overcome the sequencing depth dependency commonly seen in other DEG methods.

Our simulation experiment using preset, true-positive genes at a minimal fold change of 2, demonstrated maximal cross-platform overlaps in the DEG lists generated by two of the RNA-Seq algorithms, baySeq and DESeq, and by two microarray methods, eBayes and SAM (Table 2). These observations are consistent with our results obtained using the HT-29 experimental data. Note however, that we were not able to evaluate the Cuffdiff algorithm using the simulated dataset. When the sensitivity of all the DEG methods were also examined in our study, the results showed that baySeq performed best among all RNA-Seq algorithms evaluated, in identifying true positive genes at each 95% minimal fold-change level. This observation is consistent with a previous study in which baySeq was found superior in ranking genes by significance to be declared [35]. DESeq tails immediately after baySeq in sensitivity curves and performed comparably well at lower fold change levels (e.g. log2 fold change ~ 1.5). The microarray DEG algorithms, SAM and eBayes, were generally found less sensitive than RNA-Seq programs.

With respect to FDR evaluation, however, baySeq resulted in more false positive calls than most of the other RNA-Seq algorithms except for NOISeq, especially when the 95% minimum fold changes of true positive genes are higher (Figure 3B, **right section**). DESeq constantly results in the lowest FDR among all the RNA-Seq algorithms evaluated in the simulation experiments, indicating its superior reliability. The NOISeq showed a very poor performance on FDR evaluation curve particularly with lower 95% minimal fold change thresholds (Figure 3B, **left section**), reflecting the fact that NOISeq's DEG discerning power by comparing noise distribution against a true signal was seriously compromised when the 'true difference' is less remarkable. In practice, it is of theoretical importance to weigh more on preventing false positives than false negatives; we thus favor DESeq over Bayseq in RNA-Seq analysis as the former method controls FDR better than the latter in higher differential significance level (Figure 3B, **right section**).

Of the two microarray DEG algorithms, SAM slightly outperforms Ebayes in both sensitivity and FDR evaluation. The traditional T-test with BH correction, not surprisingly, showed a very poor performance in identifying true positives, probably due to its inappropriate independence assumption. When we view our results from the perspective of platform comparison, it is generally expected that DESeq and SAM can lead to consistent and reasonable DEG results -- an observation which is exactly reflected in our HT-29 experiment (Table 1).

Finally, to begin to address the biological significance of these studies, we undertook to validate that treatment of HT-29 colon cancer cells with 5 μM 5-Aza would relieve suppression of *SPARC *gene expression. While this anticipated outcome was confirmed using both the RNA-Seq data and qRT-PCR data, it was not observed in the microarray data. In addition a higher percentage of other DEGs identified using both platforms or RNA-Seq only was confirmed by qRT-PCR than the DEGs identified using microarray alone.

## Conclusions

A strong correlation of genomic expression profiles was observed between the microarray and RNA-Seq platforms with the latter technology detecting more genes across the genome. Remarkable differences between the two platforms in terms of (1) the existence of both fixed and proportional biases detected by the errors-in-variable (EIV) regression model, and (2) discrepancies in DEG identification have been discovered in our study. We further confirmed that the DEG discrepancies are mostly related to the different algorithms used for both platforms. Among all the DEG algorithms surveyed in this study, the largest cross-platform overlaps were observed between the DEG lists generated by two RNA-Seq algorithms, baySeq and DESeq, and the DEG lists generated by two microarray algorithms, SAM and eBayes, from the HT-29 experimental dataset. The simulation studies, which did not include evaluation of Cuffdiff, indicate that the the DESeq algorithm outperformed the other RNA-Seq algorithms, based upon the combined considerations of sensitivity and false discovery rate. DESeq also demonstrated the highest overlap rate with the DEG list generated by SAM from the microarray data. Overall, the nonparametric based DEG methods such as SAMSeq or NOISeq exhibited suboptimal performance compared to their parametric counterparts, partly due to the limited number of replicates. QRT-PCR validated a higher percentage of the DEGs identified by both platforms and RNA-Seq only, than the DEGs identified by microarray only. Finally, while there were common IPA canonical pathways identified by both microarray and RNA-Seq data, a large number of additional canonical pathways were identified by RNA-Seq data alone. No additional canonical pathways were identified by microarray data alone.

## List of abbreviations used

5-Aza: 5-azadeoxy-cytidine; EIV: Errors-In-Variables; DEG: differentially expressed genes; FDR: false discovery rate; FC: fold change; qRT-PCR: quantitative reverse transcriptase polymerase chain reaction.

## Competing interests

W.R.M. has participated in Illumina sponsored meetings over the past four years and received travel reimbursement and an honorarium for presenting at these events. Illumina had no role in decisions relating to the study/work to be published, data collection and analysis of data and the decision to publish.

W.R.M. has participated in Pacific Biosciences sponsored meetings over the past three years and received travel reimbursement for presenting at these events.

W.R.M. is a founder and shareholder of Orion Genomics, which focuses on plant genomics and cancer epigenetics.

The other authors declare that they have no competing interests.

## Authors' contributions

Drs. Jennie Williams, Eric Antinoiou, Paula Denoya and W. Richard McCombie performed the experiment; Dr. Xiao Xu designed the study, analyzed the data and wrote the initial draft of the paper; Yuanhao Zhang also designed the study and performed EIV and simulation analysis, Drs. Song Wu and Wei Zhu participated in study design and provided statistical advices, Ellen Li funded and designed the study. Dr. Nicholas Davidson performed the biological functional analyses. All authors participated in revising the final draft of the manuscript.

## Supplementary Material

Additional file 1 **Table of ABI Taqman Assay Kit IDs used in qRT-PCR assays**. The list of 13 genes selected by majority vote on platform specific DEG methods plus GAPDH and SPARC are included in this table. Click here for file

Additional file 2 **Table of DEGs for each algorithm**. In this file, each gene that was significant in at least one algorithm is shown with corresponding fold change. NA under an algorithm indicates the gene is not included in the DEG list generated by that algorithm. Up-regulated and Down-regulated genes are in separate sheets. Log2 scaled normalized gene expression intensities are shown for each gene on both microarray and RNA-Seq platform. Click here for file

Additional File 3 **Table of significant pathway for both up and down regulated DEGs**. Pathways that were at least significant in one algorithm are shown in this file. If a pathway is marked as significant in an algorithm, the corresponding DEGs detected by that algorithm are included; otherwise the cell is left blank. Both up-regulated and down-regulated pathways are shown in this file, while down-regulated pathways are bolded. Click here for file

## Declarations

Publication of this article is funded by the Stony Brook Institute for Clinical and Translational Sciences Fusion Award (P.D.), the Simons Foundation (E.L.), and NIH grants R01CA140487 (J.W.), NIH RO1DK-56260 (N.D.), RO1HL-38180 (N.D.) and P30DK-52574 (N.D.). RNA-Sequencing was performed at the Cold Spring Harbor Laboratory Genome Sequencing Core, which is partially supported by NCI center grant CA045508.

This article has been published as part of *BMC Bioinformatics *Volume 14 Supplement 9, 2013: Selected articles from the 8th International Symposium on Bioinformatics Research and Applications (ISBRA'12). The full contents of the supplement are available online at http://www.biomedcentral.com/bmcbioinformatics/supplements/14/S9.
