# Supplementary material for: Parallel comparison of Illumina RNA-Seq and Affymetrix microarray platforms on transcriptomic profiles generated from 5-aza-deoxy-cytidine treated HT-29 colon cancer cells and simulated datasets
Source: BMC Bioinformatics. 2013 Jun 28;14(Suppl 9):S1. doi: 10.1186/1471-2105-14-S9-S1 (PMC3697991; doi:10.1186/1471-2105-14-S9-S1)
Supplement: Additional file 1 — Table of ABI Taqman Assay Kit IDs used in qRT-PCR assays. The list of 13 genes selected by majority vote on platform specific DEG methods plus GAPDH and SPARC are included in this table. [file 1471-2105-14-S9-S1-S1.doc]

**Table of ABI Taqman Assay Kit IDs used in qRT-PCR assays.**

| **Gene Symbol** | **Gene** | **TaqmanAssay ID** |
| --- | --- | --- |
| *ALDH3A1* | Aldehyde dehydrogenase 3 family, member A1 | Hs00964880_m1 |
| *EFNB1* | Ephrin-B1 | Hs00270004_m1 |
| *GAPDH* | Glyceraldehyde 3-phosphate dehydrogenase | Hs02758991_g1 |
| *GGT1* | Gamma-glutamyltransferase 1 | Hs00980756_m1 |
| *GGT7* | Gamma-glutamyltransferase 7 | Hs01067375_m1 |
| *IL1R1* | Interleukin 1 receptor, type I | Hs00991002_m1 |
| *IL8* | Interleukin 8 | Hs00174103_m1 |
| *IRF7* | Interferon regulatory factor 7 | Hs01014809_g1 |
| *LIPE* | Hormone sensitive lipase | Hs00193510_m1 |
| *MAPK10* | Mitogen-activated protein kinase 10 | Hs00373461_m1 |
| *PLCL1* | Phospholipase C-like 1 | Hs00196518_m1 |
| *RPSA* | Ribosome protein SA or laminin receptor 1. | Hs03046712_g1 |
| *SPARC* | Secreted protein acidic and rich in cysteine | Hs00234160_m1 |
| *TAF11* | Transcription initiation factor TFIID subunit 11 | Hs01051507_m1 |
| *TGM2* | Transglutaminase 2 | Hs00190278_m1 |
